# Plant Heteropolysaccharides as Potential Anti-Diabetic Agents: A Review

**DOI:** 10.3390/cimb47070533

**Published:** 2025-07-09

**Authors:** Dan He, Can Cui

**Affiliations:** 1 Fisheries College, Ocean University of China, Qingdao 266003, China; 2 College of Life Science and Agroforestry, Qiqihar University, Qiqihar 161006, China; 19846339619@163.com

**Keywords:** plant heteropolysaccharides, anti-diabetic agent, mechanisms, diabetes mellitus, gut microbiota, insulin resistance

## Abstract

Diabetes mellitus (DM), a chronic metabolic disease, poses a significant challenge to global health. Although type 1 diabetes mellitus (T1DM), type 2 diabetes mellitus (T2DM), gestational diabetes mellitus (GDM), and other types of diabetes mellitus differ in pathological mechanisms, they converge in that hyperglycemia is a universal clinical hallmark. Currently, the antidiabetic medications employed in clinical practice for blood glucose management require long-term administration and are associated with various side effects that can adversely impact human health. Plant heteropolysaccharides have emerged as promising candidates for anti-diabetic therapy, owing to their abundant natural sources, absence of toxicities, and confirmed hypoglycemic activities. This review aims to summarize the anti-diabetic mechanisms of plant heteropolysaccharides by dissecting the key biological pathways associated with clinical intervention in DM, including the modulation of insulin secretion, a reduction in insulin resistance, and an alteration in the composition of the gut microbiota. For these reasons, these findings provide a theoretical framework for the clinical application of plant heteropolysaccharides and indicate that they are expected to become natural agents used in treating DM.

## 1. Introduction

Diabetes mellitus (DM) has emerged as a global epidemic chronic disease, primarily classified into type 1 diabetes mellitus (T1DM), type 2 diabetes mellitus (T2DM), gestational diabetes mellitus (GDM), and other types of DM [1,2]. The global DM population is projected to reach 783 million by 2045, posing a substantial burden on healthcare systems worldwide [3]. Pathophysiologically, T1DM is characterized by hyperglycemia resulting from the autoimmune-mediated destruction of pancreatic islet β-cells [4]. T2DM is marked by insulin resistance, manifesting as reduced insulin sensitivity that impairs glucose uptake and utilization, ultimately leading to hyperglycemia [5]. GDM occurs when excessive placental hormone secretion during pregnancy induces insulin resistance, causing elevated blood glucose [6]. Other DM types, including genetic, endocrine-related, and drug-induced DM, involve different degrees of glucose metabolism dysregulation, all culminating in hyperglycemic states [7]. Although there are diverse pathological mechanisms among different types of DM, the common feature of hyperglycemia is a crucial clinical parameter for DM diagnosis, treatment, and monitoring. Currently, therapeutic agents for DM include metformin, sulfonylureas, thiazolidinediones, glinides, glucagon-like peptide-1 (GLP-1) receptor agonists, sodium-glucose cotransporter 2 (SGLT2) inhibitors, dipeptidyl peptidase-4 (DPP-4) inhibitors, α-glucosidase inhibitors, and exogenous insulin [8,9]. While these agents are effective in managing blood glucose, they are associated with notable adverse effects, including abdominal pain, gastrointestinal disturbances (diarrhea/constipation), nausea, hypersensitivity reactions, weight fluctuations, and severe hypoglycemia, which severely affect the quality of life of patients [10,11,12,13]. This underscores the urgent need for safer therapeutic agents.

In this context, plant-derived heteropolysaccharides have garnered significant attention as potential DM interventions, driven not only by their non-toxic profile and lack of side effects but, more importantly, by their glucose-regulatory properties. Notably, while emerging mechanisms like epigenetic regulation exhibit promise in DM intervention, current research remains predominantly focused on the prevention and treatment of DM complications and remains in the exploratory stage of clinical translation [14,15]. Numerous studies have shown that plant heteropolysaccharides mediate hypoglycemic effects through the following mechanisms. Firstly, plant heteropolysaccharides regulate insulin secretion and maintain pancreatic β-cell function to exert a hypoglycemic effect [16]. Secondly, plant heteropolysaccharides modulate insulin-related signalling pathways to ameliorate insulin resistance, thereby preserving systemic glucose homeostasis [17]. Thirdly, plant heteropolysaccharides restructure the gut microbiota by promoting the enrichment of beneficial bacteria and/or inhibiting the proliferation of pathogenic bacteria, thus intervening in DM pathogenesis [18]. These mechanisms have been validated as critical intervention pathways in numerous clinical and preclinical studies [19,20,21,22]. This review provides a comprehensive overview of plant-derived heteropolysaccharides targeting DM-related intervention targets by integrating the existing literature.

The animal models selected in this review (such as mice and rats) are highly similar to human diseases in terms of the core pathophysiology of DM [23,24]. At the preclinical research stage, mechanisms can be explored using animal models to provide clear targets for subsequent clinical research [25,26]. A literature search was conducted in scientific databases (including Web of Science, Scopus, and PubMed) using keywords such as “plant,” “diabetes,” and “heteropolysaccharide” to retrieve recent English-language publications. Modern biotechnology approaches have enabled the isolation and characterization of high-purity heteropolysaccharides from edible and medicinal plants, including traditional Chinese herbs and common edible plants. However, their clinical translation remains limited, which is likely closely associated with the insufficient mechanistic dissection of their anti-diabetic effects. As a narrative review, this study systematically screened and collated reports from a substantial body of literature on plant-derived heteropolysaccharides with proven antidiabetic efficacy and their underlying action mechanisms. It not only provides scientific evidence for their clinical translation but also paves new avenues for the development of antidiabetic natural medicines from the perspective of target-based mechanism dissection.

## 2. The Intervention Mechanisms of Plant Heteropolysaccharides on Diabetes Mellitus

### 2.1. Regulation of Insulin Secretion and Pancreatic β-Cells Function

**Figure 1:**
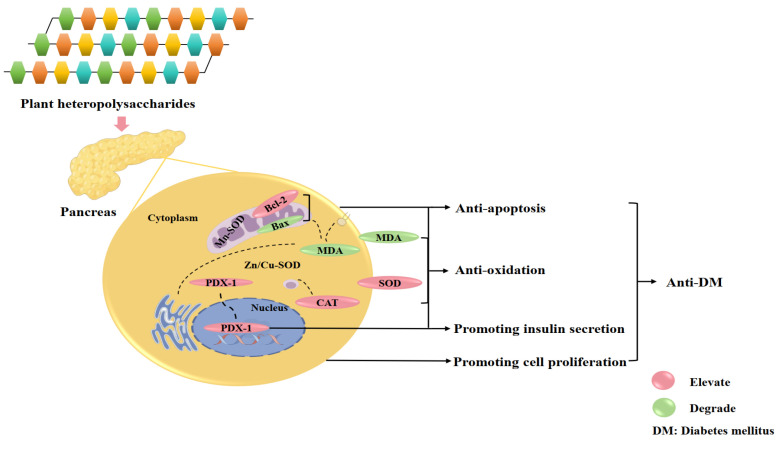
The regulation of pancreatic β-cells by plant heteropolysaccharides. This figure illustrates how plant heteropolysaccharides preserve pancreatic β-cells and the insulin secretory capacity in diabetes mellitus (DM) management. The relevant mechanisms are described as follows. Apoptosis inhibition: Plant heteropolysaccharides reduce pancreatic β-cells apoptosis by regulating the balance between pro-apoptotic protein Bax and anti-apoptotic protein Bcl-2. Promotion of proliferation: Plant heteropolysaccharides stimulate pancreatic β-cells regeneration, which is critical for maintaining insulin production. Antioxidant enhancement: Plant heteropolysaccharides enhance the activity of antioxidant enzymes (such as superoxide dismutase [SOD] and catalase [CAT]) and reduce malondialdehyde (MDA) levels, thereby protecting pancreatic β-cells from oxidative stress. PDX-1 regulation: Plant heteropolysaccharides upregulate PDX-1 (pancreatic and duodenal homeobox 1), a transcription factor essential for insulin gene expression and pancreatic β-cells function.

Pancreatic β-cells play a critical role in maintaining glucose homeostasis through insulin secretion. Therefore, protecting pancreatic β-cells and preserving their function is pivotal for DM management. Studies have demonstrated that plant heteropolysaccharides protect pancreatic β-cells via multiple pathways, including the inhibition of pancreatic β-cell apoptosis, the promotion of pancreatic β-cell proliferation, and the enhancement of cellular antioxidant defense capabilities (Figure 1). From the perspective of apoptosis regulation mechanisms, plant heteropolysaccharides effectively inhibit pancreatic β-cell apoptosis by modulating apoptosis-related proteins. For example, heteropolysaccharides from *Morus alba* L. leaf (Moraceae, *Morus*, containing arabinose, xylose, glucose, rhamnose, mannose) have been shown to reduce pancreatic β-cells apoptosis in DM rats [27]. It suppresses apoptosis by regulating proteins such as B-cell lymphoma/leukemia-2 (bcl-2) and Bcl-2-associated X protein (bax), thereby preserving both the number and function of pancreatic β-cells. In addition, *Morus alba* L. leaf heteropolysaccharides up-regulated the expression of pancreatic duodenal homeobox 1 (PDX-1), a key transcription factor for insulin gene transcription and β-cell function. PDX-1 promotes insulin synthesis and secretion by activating downstream signalling pathways, thereby enhancing β-cell functionality. Recent studies on heteropolysaccharides from *Cyclocarya paliurus* leaves (Juglandaceae, *Cyclocarya*, CP, containing xylose, arabinose, glucose, galactose, rhamnose, mannose), a traditional Chinese medicinal and edible plant, have shown that CP significantly promotes pancreatic β-cell regeneration and alleviates diabetic symptoms in DM rats. This effect is mediated by inhibiting bax protein expression, enhancing Bcl-2 protein activity, and regulating hormone secretion in pancreatic tissues [28].

In terms of cell proliferation, heteropolysaccharides from *Hovenia dulcis* (Rhamnaceae, *Hovenia*, containing arabinose, galactose, glucose) and *Lycium barbarum* L. (Solanaceae, *Lycium*, containing rhamnose, arabinose, xylose, mannose, glucose, galactose, galacturonic acid) promote pancreatic β-cell regeneration/proliferation, maintaining insulin secretion to meet the metabolic demands [29,30]. Notably, *Hovenia dulcis* heteropolysaccharides reduce fasting blood glucose levels and increase plasma insulin levels, which are closely associated with pathways regulating PDX-1 expression. Through these pathways, they restore the apoptosis–regeneration balance of pancreatic β-cells, thereby repairing impaired pancreatic β-cell function in a T1DM rat model [29]. In addition, the heteropolysaccharide from *Coptis chinensis* (Ranunculaceae, *Coptis*, comprising glucose, arabinose, xylose, galactose, and galacturonic acid) also enhances antioxidant enzyme activities (e.g., superoxide dismutase [SOD] and catalase [CAT]) and reduces malondialdehyde (MDA) levels, thereby augmenting the antioxidant defense system of pancreatic β-cells and preserving their functional integrity [31]. As shown in Table S1, the extraction processes of the above active plant heteropolysaccharides mainly include hot water extraction or the hot water extraction–ethanol precipitation method. It is worth noting that studies have shown that the biological activities of polysaccharides are determined by their structural characteristics [32,33]. Consequently, to preserve their bioactivity, the structure–activity relationships underlying the antidiabetic effects of these plant heteropolysaccharides necessitate further validation in the aforementioned models. Nevertheless, these findings underscore the multifaceted regulatory roles of plant heteropolysaccharides in insulin secretion and β-cell homeostasis, proffering compelling evidence for their translational potential in DM therapy.

### 2.2. Improvement of Insulin Resistance

**Figure 2:**
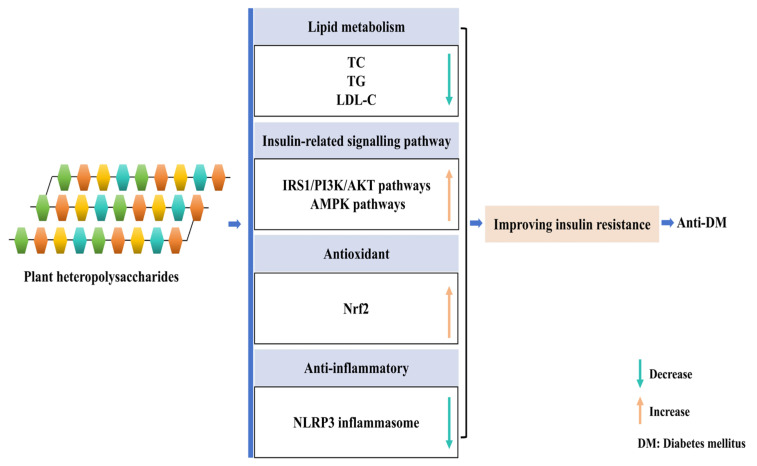
Improvement in insulin resistance by plant heteropolysaccharides. Plant heteropolysaccharides improve insulin resistance through the following mechanisms: activating insulin signaling pathways related to insulin receptor substrate 1/phosphatidylinositol 3-kinase/protein kinase B (IRS1/PI3K/AKT) and AMP-activated protein kinase (AMPK) pathways, triggering nuclear factor erythroid 2-related factor 2 (Nrf2)-mediated antioxidant defense pathways, inhibiting nucleotide-binding oligomerization domain-like receptor family pyrin domain containing 3 (NLRP3) inflammasome, and reducing lipid accumulation.

In individuals with DM, insulin resistance is characterized by reduced insulin sensitivity, leading to impaired glucose uptake and utilization, which ultimately contribute to hyperglycemia. In recent years, studies based on animal models have demonstrated that plant heteropolysaccharides ameliorate insulin resistance through different mechanisms (Figure 2). Firstly, plant heteropolysaccharides enhance insulin sensitivity by regulating critical signalling pathways. For example, *Momordica charantia* L. heteropolysaccharide (Cucurbitaceae, *Momordica*, containing rhamnose, glucuronic acid, galacturonic acid, glucose, galactose, arabinose) and *Polygonatum sibiricum* heteropolysaccharide (Asparagaceae, *Polygonatum*, containing fructose, glucose) activate the insulin receptor substrate 1/phosphatidylinositol 3-kinase/protein kinase B (IRS1/PI3K/AKT) and AMP-activated protein kinase (AMPK) pathways to improve insulin sensitivity [34,35]. In a T2DM mice model, *Momordica charantia* L. heteropolysaccharide significantly reduced fasting serum glucose, improved glucose tolerance, and alleviated insulin resistance by activating these signaling pathways [34]. Xie et al. [35] further showed that *Polygonatum sibiricum* heteropolysaccharides restore insulin sensitivity by upregulating PI3K and AKT phosphorylation while downregulating forkhead box protein O1 (FoxO1) and glycogen synthase kinase 3β (GSK3β) phosphorylation, thereby reinstating the insulin-mediated PI3K/AKT signaling axis in T2DM mice. These pathways form the central regulatory network of glucose homeostasis and have long been established as classical therapeutic targets in DM, underscoring their role as pivotal determinants of plant heteropolysaccharide action [36,37].

Secondly, oxidative stress and inflammation are fundamental drivers of insulin resistance, both of which are targeted by plant heteropolysaccharides. The *Codonopsis lanceolata* heteropolysaccharide (Campanulaceae, *Codonopsis*, CLPS, containing rhamnose, arabinose, xylose, mannose, galactose, glucose, galacturonic acid, glucuronic acid) ameliorates insulin resistance in high-fat/high-sucrose-fed mice by activating the Nrf2-mediated antioxidant defense system [38]. This is evidenced by reduced MDA levels, elevated reduced glutathione (GSH)/oxidized glutathione (GSSG) ratios, and the enhanced expression of antioxidant enzymes (e.g., SOD, CAT). Concurrently, *Bletilla striata* heteropolysaccharides (Orchidaceae, *Bletilla*, BSP, containing mannose, glucose) enhance insulin sensitivity by suppressing NLRP3 inflammasome-driven inflammation [39]. In a diabetic foot ulcer (DFU) model induced by a high-fat diet and low-dose streptozotocin (STZ), BSP promoted wound healing by inhibiting NLRP3 inflammasome activation and reducing interleukin-1β (IL-1β) secretion, thereby restoring local insulin responsiveness. These findings highlight the dual role of plant heteropolysaccharides in targeting both nuclear factor erythroid 2-related factor 2 (Nrf2)-dependent antioxidant pathways and nucleotide-binding oligomerization domain-like receptor family pyrin domain containing 3 (NLRP3)-mediated inflammatory responses to intervene in insulin resistance.

Finally, plant heteropolysaccharides also address the intimate link between insulin resistance and dyslipidemia by regulating lipid metabolism. Heteropolysaccharides isolated from *Pueraria lobata* root (Fabaceae, *Pueraria*, containing glucose), *Gynura divaricata* (L.) DC (Asteraceae, *Gynura*, containing galacturonic acid, xylose, galactose, glucose, rhamnose, arabinose, fucose, glucuronic acid), *Dioscorea opposita* (Dioscoreaceae, *Dioscorea*, containing mannose, glucose, galacturonic acid, galactose, glucuronic acid), *Juglans regia* L. green husk (Juglandaceae, *Juglans*, containing glucuronic acid, arabinose, galactose), and *Zizyphus jujube cv. Shaanbeitanzao* (Rhamnaceae, *Zizyphus*, containing arabinose, galactose, galacturonic acid) consistently reduce the serum total cholesterol (TC), triglycerides (TG), and low-density lipoprotein cholesterol (LDL-C) in animals [40,41,42,43,44]. This confirms that plant heteropolysaccharides enhance insulin sensitivity by inhibiting lipid accumulation.

Notably, among the investigated plant heteropolysaccharides, only those from *Pueraria lobata* root and *Gynura divaricata* (L.) DC were extracted using cold-water extraction and ultrasound-assisted water extraction, respectively, while others followed the conventional hot-water extraction or alcohol precipitation protocols (Table S1). Collectively, these findings highlight the diversity of polysaccharide extraction methods, which not only drive technological innovation in extraction processes but also significantly enhance the yield of bioactive polysaccharides. The observed diversity in extraction approaches paves the way for the further optimization of polysaccharide isolation technologies, ultimately contributing to a higher yield and purity in large-scale production. Current research indicates that polysaccharides exhibit structure–activity relationships; therefore, beyond polysaccharide yield and purity, attention should be directed toward the stability of extraction processes to preserve their structural consistency [45]. Whether the antidiabetic activities of these plant heteropolysaccharides are influenced by their structural features remains unclear, warranting further verification. In conclusion, plant heteropolysaccharides improve insulin resistance in DM through diverse mechanisms, including the direct activation of the insulin signalling pathway and indirect effects via antioxidant, anti-inflammatory, and lipid regulatory actions. These properties highlight their therapeutic potential for the prevention and treatment of DM.

### 2.3. Modulation of the Gut Microbiota

The gut microbiota has emerged as a crucial factor in the pathogenesis of DM, positioning it as a potential biomarker for DM diagnosis [46,47,48]. Multi-omics studies have revealed significant dysbiosis in the gut microbiota of DM, characterized by a reduced abundance of beneficial bacteria and the aberrant proliferation of conditionally pathogenic species [49,50]. Plant heteropolysaccharides, a class of natural compounds composed of various monosaccharides, effectively reverse gut microbiota dysbiosis in DM models, especially in T2DM animal models (Table 1). Heteropolysaccharides from green tea, Fu brick tea, Yellow leaves of Wuyi rock tea, red kidney bean, *Astragalus membranaceus*, *Glycyrrhiza uralensis* seeds, *Apocynum venetum* leaves, *Cucurbita pepo* ‘lady godiva’, *Achyranthes bidentata*, *Chenopodium quinoa* Willd., Blackberry, *Rosa roxburghii tratt* fruit, *Sargassum fusiforme*, *Lycium barbarum* L., *Dendrobium officinale*, *Dendrobium officinale* leaf, *Ulva lactuca*, *Macrocystis pyrifera*, *Fucus vesiculosus*, *Berberis dasystachya*, *Coix* seed, *Fructus mori*, *Polygonum cuspidatum*, *Laminaria japonica*, *Cyclocarya paliurus*, *Sarcandra glabra*, *Citrus unshiu* Marc., and *Psidium guajava* L. exert hypoglycemic effects by increasing the abundance of beneficial flora and/or decreasing the abundance of pathogenic flora in diabetic rats or mice [51,52,53,54,55,56,57,58,59,60,61,62,63,64,65,66,67,68,69,70,71,72,73,74,75,76,77,78,79,80,81,82]. For example, heteropolysaccharides from *Glycyrrhiza uralensis* seeds significantly increased the abundances of *Akkermansia*, *Lactobacillus*, *Romboutsia*, and *Faecalibaculum* (i.e., probiotic flora) in high-fat diet/streptozotocin-induced type 2 diabetic mellitus mice, while reducing the populations of *Escherichia-Shigella* and *Clostridium sensu stricto 1* (i.e., pathogenic flora), thereby exhibiting hypoglycemic, hypolipidemic, antioxidant, and anti-inflammatory activities [56]. In an identical animal model, *Psidium guajava* L. heteropolysaccharides inhibited pathogenic flora, including *Bilophila*, *Desulfovibrio*, and Uncultured_f_*Desulfovibrionaceae*, while promoting the proliferation of beneficial bacteria such as *Bifidobacterium* and *Bacteroides*, thus exerting hypoglycemic, hypolipidemic, and anti-inflammatory effects [82]. This indicates that heteropolysaccharides from different sources and with different compositions exhibit distinct efficacy in intervening in T2DM within the same animal model.

**Figure 3:**
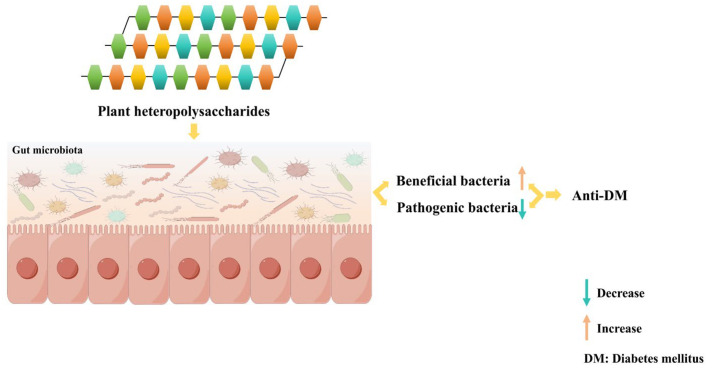
Modulation of the gut microbiota by plant heteropolysaccharides. Plant heteropolysaccharides exert their anti-diabetes mellitus (DM) effects by regulating the gut microbiota, a process that involves the enrichment of beneficial bacteria and a reduction in pathogenic bacteria.

Notably, heteropolysaccharides derived from the same plant source exhibit distinct biological activities due to differences in their monosaccharide compositions (Table 1 and Table S2). For instance, the heteropolysaccharide from *Hizikia fusifarme* (comprising fucose, mannose, rhamnose, glucose, xylose, and glucuronic acid) increased the abundances of *Ruminococcaceae*, *Turicibacter*, *Faecalibaculum*, *Mollicutes*_RF39-norank, *Lachnospiraceae*_NK4A136_group, and *Lactobacillus* in high-sugar and high-fat-diet/STZ-induced T2DM rats [63]. In contrast, the heteropolysaccharide from *Sargassum fusiforme* (containing fucose, mannose, galactose, glucose, rhamnose, xylose, and glucuronic acid) enhanced the abundances of *Ruminococcaceae*, *Mollicutes*_RF39-norank, and *Prevotellaceae*_NK3B31_group in the same model [64]. It is speculated that differences in the increased microbial abundances lead to distinct biological activities. In addition to hypoglycemic effects, the heteropolysaccharide from *Hizikia fusifarme* exhibits hypolipidemic, antioxidant, and anti-inflammatory activities, whereas that from *Sargassum fusiforme* shows hypolipidemic and antioxidant effects. Additionally, as shown in Table 1 and Table S1, heteropolysaccharides from different plant sources have demonstrated antidiabetic effects. However, no regular patterns in their efficacy have been identified in terms of plant taxonomy, heteropolysaccharide composition, or animal models, and numerous factors affect their efficacy. Notably, different plant-derived heteropolysaccharides exhibit specific regulatory effects on the gut microbiota, manifested as targeted changes in the abundance of specific microbial taxa. For example, *Cyclocarya paliurus* heteropolysaccharide regulates a broader range of gut microbiota in T2DM rats, including *Ruminococcus bromii*, *Anaerotruncus colihominis*, *Clostridium methylpentosum*, *Rosebui ia intestinalis*, *Roseburia hominis*, *Clostridiumasparagiforme*, *Pseudoflavonifractorcapillosus*, *Intestinimonasbutyriciproducens*, *Intestinimonas*_sp._GD2, *Oscillibacter valericigenes*, and *Oscillibacter ruminantium* [79]. In contrast, *Sarcandra glabra* heteropolysaccharide enriches Bacteroidales S24-7 in mice with diabetes and a spontaneous mutation, which are beneficial bacteria with hypoglycemic effects [80]. Additionally, in addition to their hypoglycemic effects, plant heteropolysaccharides exhibit hypolipidemic, antioxidant, and anti-inflammatory activities. Currently, the extraction methods for plant heteropolysaccharides affecting the gut microbiota of animals comprise hot-water extraction–alcohol precipitation, multi-enzymatic synergistic hydrolysis–alcohol precipitation, hot water/alkaline solution extraction–alcohol precipitation, ultrasound-assisted water extraction–alcohol precipitation, ultrasound-assisted dual-enzymatic hydrolysis–ethanol precipitation, water extraction–alcohol precipitation, and ammonium sulfate–tert–butanol three-phase partitioning (Table S1). Notably, water extraction–alcohol precipitation emerges as the most prevalently adopted protocol among these methods. Collectively, these findings indicate that heteropolysaccharides from distinct plant sources exhibit the differential regulation of beneficial and pathogenic flora. Based on this differential regulatory property of plant heteropolysaccharides regarding the gut microbiota, a strategy of combined intervention with multiple heteropolysaccharides is proposed for DM (Figure 3). This synergistic model enables the regulation of multiple key microbial communities, the systematic optimization of the composition of the gut microbiota, and the efficient restoration of microbiota homeostasis, thus offering a novel approach for DM prevention and treatment. Additionally, studies have shown that the regulatory effects on beneficial and pathogenic bacteria in the gut are also associated with the structure of polysaccharides [83]. Therefore, the structure–activity relationships of these plant heteropolysaccharides should be further clarified in future applications.

## 3. Conclusions and Perspectives

Plant heteropolysaccharides demonstrate pleiotropic regulatory effects on hyperglycemia in vivo, primarily through modulating insulin secretion, preserving pancreatic β-cell integrity, ameliorating peripheral insulin resistance, and reprogramming the composition of the gut microbiota. This highlights the pharmacological potential of plant heteropolysaccharides in intervening with both the onset and progression of DM. Currently, these plant heteropolysaccharides are found in *Camellia*, *Phaseolus*, *Hovenia*, *Astragalus*, *Glycyrrhiza*, *Apocynum*, *Cucurbita*, *Achyranthes*, *Rubus*, *Rosa*, *Sargassum*, *Lycium*, *Dendrobium*, *Ulva*, *Macrocystis*, *Fucus*, *Berberis*, *Coix*, *Morus*, *Polygonum*, *Gynura*, *Laminaria*, *Cyclocarya*, *Sarcandra*, *Bletilla*, *Coptis*, *Citrus*, *Codonopsis, Momordica, Pueraria*, *Dioscorea*, *Zizyphus*, *Juglans*, and *Psidium*. Despite the encouraging findings regarding the antidiabetic properties of plant heteropolysaccharides, numerous research gaps warrant systematic investigation. While researchers have elucidated certain signaling pathways, they have yet to fully decipher the intricate molecular mechanisms; in particular, the interactions between different signaling pathways and the roles of specific heteropolysaccharide structures in these processes remain to be further explored. For instance, the precise binding modes of plant heteropolysaccharides to molecular targets within the insulin signaling axis, and how structural modifications to these plant heteropolysaccharides influence their biological activities, merit in-depth investigation. Additionally, whether species-specific disparities between animal models and human DM may attenuate the therapeutic efficacy of plant heteropolysaccharides in humans necessitates rigorous clinical evaluation. In future clinical trials, the efficacy, safety, and optimal dosage regimen of these heteropolysaccharides should be comprehensively evaluated, while their long-term effects and potential adverse reactions should also be explored. In terms of translational development, considerable efforts should be invested in the formulation of antidiabetic therapeutics based on plant heteropolysaccharides. This necessitates the optimization of extraction, purification, and formulation technologies to ensure their stability, bioavailability, and therapeutic consistency. Furthermore, investigating the combinatorial use of plant heteropolysaccharides with existing antidiabetic agents could offer novel strategies for more efficacious DM management.

## Figures and Tables

**Table 1:** Gut microbiota as an antidiabetic target for plant heteropolysaccharides.

Source	Experimental Models	Gut Microbiota	Function	References
Green tea	High fat diet combined with streptozotocin induced type 2 diabetic mellitus rats	Restored the relative abundance of *Lachnospira*, *Victivallis*, *Roseburia*, and *Fluviicola*	Hypoglycemic and hypolipidemic effects	[51]
Fu brick tea	High-fat diet/streptozotocin-induced type 2 diabetic mellitus rats	Increased the abundance of *Ruminococcus*, *Lactobacillus*, and *Lachnospiraece*_NK4A136_group; Reduced the abundance of *Prevotella* and *Faecalibaculum*	Hypoglycemic, hypolipidemic, and antioxidant effects	[52]
Yellow leaves of Wuyi rock tea	Streptozotocin-induced type 2 diabetic mellitus rats	Increased the abundance of *Bifidobacterium*, *Blautia*, *Dorea*, and *Oscillospira*; Decreased the abundance of *Desulfovibrio* and *Lactobacillus*	Hypoglycemic effect	[53]
Red kidney bean	Streptozotocin-induced type 2 diabetic rats	Enriched to *Bacteroides*, *Phascolarctobacterium*, *Succinivibrio*, and *Blautia*	Hypoglycemic and hypolipidemic effects	[54]
*Astragalus membranaceus*	Diabetic *db/db* mice	Increased the abundance of *Akkermansia* and *Faecalibaculum*	Hypoglycemic effect	[55]
*Glycyrrhiza uralensis* seeds	High-fat diet/streptozotocin-induced type 2 diabetic mellitus mice	Increased the abundances of *Akkermansia*, *Lactobacillus*, *Romboutsia*, and *Faecalibaculum*; Decreased the abundances of *Escherichia-Shigella*, and *Clostridium sensu stricto 1*	Hypoglycemic, hypolipidemic, antioxidant, and anti-inflammatory effects	[56]
*Apocynum venetum* leaves	High-fat diet and streptozocin-induced type 2 diabetic mice	Increased the abundance of *Odoribacter*, *Anaeroplasma*, *Parasutterella*, and *Muribaculum*; Decreased the abundance of *Enterococcus*, *Klebsiella*, and *Aerococcus*	Hypoglycemic and hypolipidemic effects	[57]
*Cucurbita pepo* ‘lady godiva’	High-fat diet induced type 2 diabetic rats	Enriched to *Bacteroidetes*, *Prevotella*, *Deltaproteobacteria*, *Oscillospira*, *Veillonellaceae*, *Phascolarctobacterium*, *Sutterella*, and *Bilophila*	Hypoglycemic and hypolipidemic effects	[58]
*Achyranthes bidentata*	High-sugar and high-fat diet/streptozotocin-induced type 2 diabetic mellitus mice	Increased the abundance of *Alloprevotella*, *Bacteroides*, *Prevotellaceae*_UCG_001, *Prevotellaceae*_NK3B31_group, and *Akkermansia*	Hypoglycemic effect	[59]
*Chenopodium quinoa* Willd.	High-fat diet and streptozocin-induced type 2 diabetic mice	Decreased the abundance of norank_f_*Muribaculaceae* and *Lachnospiraceae*_NK4A136_group; Increased the relative abundance of *Akkermansia*, unclassified_f_*Lachnospiraceae*, norank_f_*Eubacterium*_*coprostanoligenes*_group, unclassified_f_*Atopobiaceae*, and norank_f_*Lachnospiraceae*	Hypoglycemic, hypolipidemic, and antioxidant effects	[60]
Blackberry	High-fat diet and streptozocin-induced type 2 diabetic mice	Increased the abundance of *Oscillospira*, *Bacteroidaceae*, *Bacteroides*; Decreased the abundance of *Allobaculum*	Hypoglycemic effect	[61]
*Rosa roxburghii tratt* fruit	Type-2 diabetic *db*/*db* mice	Increased the abundances of *Bacteroidaceae*, *Bacteroidaceae* S24-7 group, and *Lactobacillaceae*	Hypoglycemic and hypolipidemic effects	[62]
*Hizikia fusifarme*	High-sugar and high-fat diet/streptozotocin-induced type 2 diabetic mellitus rats	Increased the abundance of *Ruminococcaceae*, *Mollicutes*_RF39-norank, *Lachnospiraceae*_NK4A136_group, *Turicibacter*, *Faecalibaculum*, and *Lactobacillus*; Decreased the abundance of *Escherichia-Shigella*	Hypoglycemic, hypolipidemic, antioxidant, and anti-inflammatory effects	[63]
*Sargassum fusiforme*	High-sugar and high-fat diet/streptozotocin-induced type 2 diabetic mellitus rats	Increased the abundance of *Ruminococcaceae*, *Mollicutes*_RF39-norank, and *Prevotellaceae*_NK3B31_group; Decreased the abundance of *Escherichia-Shigella*	Hypoglycemic, hypolipidemic, and antioxidant effects	[64]
*Lycium barbarum*	High fat diet combined with streptozotocin induced type 2 diabetic mellitus rats	Increased the abundance of *Bacteroides*, *Ruminococcaceae*_UCG-014, *Intestinimonas*, *Mucispirillum*, and *Ruminococcaceae*_UCG-009; Decreased the abundance of *Allobaculum*, *Dubosiella*, and *Romboutsia*	Hypoglycemic and hypolipidemic effects	[65]
*Lycium barbarum* L.	High-fat diet-induced diabetic mice	Increased the abundance of *Allobaculum* and *Romboutsia*	Hypoglycemic effect	[66]
*Dendrobium officinale*	High-sugar and high-fat diet/streptozotocin-induced prediabetic rice	Enriched to *Roseburia, Bifidobacterium, Lactobacillus, Alloprevotella*, and *Bacteroides*	Hypoglycemic effect	[67]
*Dendrobium officinale*	High-fat diet and streptozocin-induced type 2 diabetic mice	Inhibited the abundance of *Helicobacter*; Facilitated the proliferation of *Allobaculum*, *Bifidobacterium*, and *Lactobacillus*	Hypoglycemic, antioxidant, and anti-inflammatory effects	[68]
*Dendrobium officinale* leaf	High fat diet combined with streptozotocin induced type 2 diabetic mellitus mice	Increased the abundance of *Lactobacillus*, *Bifidobacterium*, and *Akkermansia*	Hypoglycemic and hypolipidemic effects	[69]
*Ulva lactuca*	High-sugar and high-fat diet/D-galactose and streptozotocin-induced aging type 2 diabetic mice	Increased the abundance of *Alloprevotella* and *Pediococcus*	Hypoglycemic effect	[70]
*Macrocystis pyrifera*	High-sugar and high-fat diet/streptozotocin-induced type 2 diabetic mellitus rats	Increased the abundance of *Muribaculaceae*_norank, *Akkermansia*, *Bifidobacterium*, *Lactobacillus*, *Olsenella*, *Lachnospiraceae*_NK4A136_group, *Ruminococcaceae*_UCG-014, *Ruminococcus*_1, *Eubacterium*_coprostanoligenes_group, and *Ruminococcaceae*_UCG-014; Decreased the abundance of *Escherichia-Shigella*	Hypoglycemic and hypolipidemic effects	[71]
*Fucus vesiculosus*	High-fat diet and streptozotocin-induced type 2 diabetic mellitus rats	Increased the abundance of *Lactobacillus*, *Muribaculaceae*_norank, *Lachnospiraceae*_Nk4A136_group, and *Bacteroides*; Reduced the abundance of *Escherichia-Shigella*, *Herminiimonas*, *Citrobacter*, and *Pseudomonas*	Hypoglycemic, hypolipidemic, and antioxidant effects	[72]
*Berberis dasystachya*	High-fat diet/streptozotocin-induced type 2 diabetic mellitus rats	Enriched to *Ruminococcaceae* NK4A214 group, *Ruminococcus 2*, *Prevotellaceae* NK3B31 group, *Eubacterium coprostanoligenes* group, *Romboutsia*, and *Alloprevotella*	Hypoglycemic and antioxidant effects	[73]
*Coix* seed	High-fat diet and streptozotocin-induced type 2 diabetic mellitus mice	Increased the abundance of *Lactobacillus*, *Akkermansia*, *Bacteroides*, and *Bifidobacterium*	Hypoglycemic effect	[74]
*Fructus mori*	Obese diabetic *db*/*db* mice	Enriched to *Bacteroidales*, *Lactobacillus*, *Allobaculum*, *Bacteroides*, and *Akkermansia*	Hypoglycemic, hypolipidemic, and antioxidant effects	[75]
*Fructus mori*	High-fat diet and streptozotocin-induced type 2 diabetic mellitus mice	The inhibition of *Shigella* and the restoration of *Allobaculum* and *Bifidobacterium*	Hypoglycemic, hypolipidemic, antioxidant, and anti-inflammatory effects	[76]
*Polygonum cuspidatum*	High-fat diet and streptozotocin-induced type 2 diabetic mellitus mice	Upregulated the population of *Lactobacillus* and *Akkermansia*	Hypoglycemic effect	[77]
*Laminaria japonica*	High-sugar and high-fat diet/streptozotocin-induced type 2 diabetic rats	Increased the abundance of *Bacteroidia*, *Campylobacteria*, *Clostridia*, *Gammaproteobacteria*, *Negativicutes*, and *Verrucomicrobi*	Hypoglycemic and hypolipidemic effects	[78]
*Cyclocarya paliurus*	High-fat diet and streptozotocin-induced type 2 diabetic rats	Increased the abundances of *Ruminococcus bromii*, *Anaerotruncus colihominis*, *Clostridium methylpentosum*, *Rosebui ia intestinalis*, *Roseburia hominis*, *Clostridiumasparagiforme*, *Pseudoflavonifractorcapillosus*, *Intestinimonasbutyriciproducens*, *Intestinimonas*_sp._GD2, *Oscillibacter valericigenes*, and *Oscillibacter ruminantium*	Hypoglycemic and hypolipidemic effects	[79]
*Sarcandra glabra*	Diabetes spontaneous mutation mice (Leptin receptor-deficient, Leprdb/db)	Enriched to *Bacteroidales* S24-7	Hypoglycemic effect	[80]
*Citrus unshiu* Marc.	Diabetic C57BL/KsJ-db/db mice	Increased the abundance of *Ligilactobacillus*, *Lactobacillus*, and *Limosilactobacillus*	Hypoglycemic and hypolipidemic effects	[81]
*Psidium guajava* L.	High-fat diet and streptozotocin-induced type 2 diabetic mice	Inhibited Uncultured_f_*Desulfovibrionaceae*, *Bilophila*, and *Desulfovibrio*; Promoted the proliferation of *Bifidobacterium* and *Bacteroides*	Hypoglycemic, hypolipidemic, and anti-inflammatory effects	[82]

## Supplementary Materials

The following supporting information can be downloaded at: https://www.mdpi.com/article/10.3390/cimb47070533/s1.
