# Supplementary material for: Plant Heteropolysaccharides as Potential Anti-Diabetic Agents: A Review
Source: Curr Issues Mol Biol. 2025 Jul 9;47(7):533. doi: 10.3390/cimb47070533 (PMC12294070; doi:10.3390/cimb47070533)
Supplement: Supplementary file 1 [file cimb-47-00533-s001.zip › cimb-3719094-supplementary.pdf]

current issues in molecular biology

MDPI

Supplementary Table S1. Extraction methods of plant heteropolysaccharides.

Source Name	Extraction Methods	References
Morus alba L. leaf	Hot water extraction-alcohol precipitation	[27]
Cyclocarya paliurus leaves	Hot water extraction	[28]
Hovenia dulcis	Hot water extraction	[29]
Lycium barbarum L.	Hot water extraction-alcohol precipitation	[30]
Coptis Chinensis	Hot water extraction	[31]
Momordica charantia L.	Thermo-ultrasonic-assisted water extraction-ethanol precipitation	[34]
Polygonatum sibiricum	Hot water extraction	[35]
Codonopsis lanceolata	Hot water extraction-alcohol precipitation	[38]
Bletilla striata	Hot water extraction-alcohol precipitation	[39]
Pueraria lobata root	Cold water extraction method	[40]
Gynura divaricata (L.) DC	Ultrasound-assisted water extraction	[41]
Dioscorea opposita	Hot water extraction-alcohol precipitation	[42]
Juglans regia L. green husk	Hot water extraction-alcohol precipitation	[43]
Zizyphus jujube cv. Shaanbeitanzao	Water extraction-alcohol precipitation	[44]
Green tea	Hot water extraction-alcohol precipitation	[51]
Fu brick tea	Hot water extraction-alcohol precipitation	[52]
Yellow leaves of Wuyi rock tea	Hot water extraction-alcohol precipitation	[53]
Red kidney bean	Multi-enzymatic synergistic hydrolysis-alcohol precipitation method	[54]
Astragalus membranaceus	Hot water extraction-alcohol precipitation	[55]
Glycyrrhiza uralensis seeds	Hot water extraction-alcohol precipitation	[56]
Apocynum venetum leaves	Hot water/alkaline solution extraction-alcohol precipitation	[57]
Cucurbita pepo 'lady godiva'	Hot water extraction-alcohol precipitation	[58]

Achyranthes bidentata	Hot water extraction-alcohol precipitation	[59]
Chenopodium quinoa Willd.	Ultrasound-assisted water extraction-alcohol precipitation method	[60]
Blackberry	Hot water extraction	[61]
Rosa roxburghii tratt fruit	Hot water extraction	[62]
Hizikia fusifarme (a synonym of *Sargassum fusiforme*)	Hot water extraction-alcohol precipitation	[63]
Sargassum fusiforme	Ultrasound-assisted dual-enzymatic hydrolysis-ethanol precipitation method	[64]
Lycium barbarum	Ultrasound-assisted water extraction-alcohol precipitation method	[65]
Lycium barbarum L. (Same as above, full Latin name format)	Hot water extraction-alcohol precipitation	[66]
Dendrobium officinale	Hot water extraction-alcohol precipitation	[67]
Dendrobium officinale	Hot water extraction-alcohol precipitation	[68]
Dendrobium officinale leaf	Hot water extraction-alcohol precipitation	[69]
Ulva lactuca	Hot water extraction-alcohol precipitation	[70]
Macrocystis pyrifera	Hot water extraction-alcohol precipitation	[71]
Fucus vesiculosus	Hot water extraction-alcohol precipitation	[72]
Berberis dasystachya	Ultrasound-assisted water extraction-alcohol precipitation method	[73]
Coix seed	Hot water extraction-alcohol precipitation	[74]
Fructus mori	Hot water extraction-alcohol precipitation	[75]

Fructus mori	Water extraction-alcohol precipitation	[76]
Polygonum cuspidatum	Hot water extraction	[77]
Laminaria japonica	Hot water extraction	[78]
Cyclocarya paliurus	Hot water extraction-alcohol precipitation	[79]
Sarcandra glabra	Hot water extraction-alcohol precipitation	[80]
Citrus unshiu Marc.	Alcohol precipitation	[81]
Psidium guajava L.	Ammonium sulfate-tert-butanol three-phase partitioning method	[82]

Supplementary Table S2. Monosaccharide composition and classification of plant heteropolysaccharides targeting gut microbiota.

Family Name	Genus Name	Source Name	Monosaccharide Composition	References
Theaceae	*Camellia*	Green tea	Rhamnose, ribose, arabinose, mannose, glucose, galactose	[51]
		Fu brick tea	Arabinose, rhamnose, galactose, mannose, galacturonic acid	[52]
		Yellow leaves of Wuyi rock tea	Arabinose, glucose, rhamnose, galactose, galacturonic acid	[53]
Fabaceae	*Phaseolus*	Red kidney bean	Fucose, arabinose, galactose, glucose, xylose, galacturonic acid	[54]
	Astragalus	*Astragalus membranaceus*	Mannose, rhamnose, galacturonic acid, glucose, galactose, arabinose	[55]
	Glycyrrhiza	*Glycyrrhiza uralensis* seeds	Galactose, mannose, xylose, glucose	[56]
Apocynaceae	*Apocynum*	*Apocynum venetum* leaves	Mannose, rhamnose, glucuronic acid, galacturonic acid, glucose, galactose, xylose, arabinose	[57]
Cucurbitaceae	*Cucurbita*	*Cucurbita pepo* 'lady godiva'	Glucose, mannose, galactose, fucose	[58]
Amaranthaceae	*Achyranthes*	*Achyranthes bidentata*	Mannose, ribose, rhamnose, glucuronic acid, galacturonic acid, glucose, galactose, arabinose, fucose	[59]
		Chenopodium quinoa Willd.	Glucose, galactose, arabinose	[60]
Rosaceae	*Rubus*	Blackberry	Arabinose, galactose, glucose, galacturonic acid, glucuronic acid	[61]
	Rosa	*Rosa roxburghii tratt* fruit	Arabinose, galactose, glucose, mannose, xylose, fucose	[62]

Sargassaceae	*Sargassum*	*Hizikia fusifarme* (a synonym of *Sargassum fusiforme*)	Fucose, mannose, rhamnose, glucose, xylose, glucuronic acid	[63]
		Sargassum fusiforme	Fucose, mannose, galactose, glucose, rhamnose, xylose, glucuronic acid	[64]
Solanaceae	*Lycium*	*Lycium barbarum*	Rhamnose, arabinose, xylose, mannose, glucose, galactose, glucuronic acid, galacturonic acid	[65]
		Lycium barbarum L. (Same as above, full Latin name format)	Rhamnose, galacturonic acid, glucose, galactose, arabinose	[66]
Orchidaceae	*Dendrobium*	*Dendrobium officinale*	Mannose, glucose	[67]
		Dendrobium officinale	Mannose, glucose, galacturonic acid, galactose, arabinose, glucuronic acid	[68]
		Dendrobium officinale leaf	Glucose, mannose, glucuronic acid, galactose	[69]
Ulvaceae	*Ulva*	*Ulva lactuca*	Rhamnose, glucose, galactose, xylose	[70]
Lessoniaceae	*Macrocystis*	*Macrocystis pyrifera*	Fucose, mannose, rhamnose, glucose, galactose, xylose, glucuronic acid	[71]
Fucaceae	*Fucus*	*Fucus vesiculosus*	Mannose, rhamnose, glucuronic acid, glucose, galactose, xylose, fucose	[72]
Berberidaceae	*Berberis*	*Berberis dasystachya*	Fucose, arabinose, galactose, glucose, xylose, mannose, galacturonic acid, guluronic acid, glucuronic acid	[73]
Poaceae	*Coix*	*Coix* seed	Fucose, rhamnose, arabinose, galactose, glucose, xylose, mannose, fructose, ribose, galacturonic acid, guluronic acid, glucuronic acid, mannuronic acid	[74]
Moraceae	*Morus*	*Fructus mori*	Arabinose, galactose, glucose, rhamnose, galacturonic acid	[75]
		Fructus mori	Arabinose, rhamnose, mannose, galactose, glucose, galacturonic acid, glucuronic acid	[76]
Polygonaceae	*Polygonum*	*Polygonum cuspidatum*	Fucose, rhamnose, arabinose, galactose, glucose	[77]
Laminariaceae	*Laminaria*	*Laminaria japonica*	Mannose, glucosamine, rhamnose, glucuronic acid, glucose, galactose, xylose, fucose	[78]
Juglandaceae	*Cyclocarya*	*Cyclocarya paliurus*	Glucose, arabinose, galactose, mannose, xylose, rhamnose, galacturonic acid, glucuronic acid, fucose, ribose	[79]

Chloranthaceae	*Sarcandra*	*Sarcandra glabra*	Fucose, rhamnose, arabinose, galactose, glucose, mannose, xylose, galacturonic acid, glucuronic acid	[80]
Rutaceae	*Citrus*	*Citrus unshiu* Marc.	Rhamnose, arabinose, galactose, galacturonic acid	[81]
Myrtaceae	*Psidium*	*Psidium guajava* L.	Arabinose, galactose, galacturonic acid, glucose, xylose, rhamnose, glucuronic acid, mannose, fucose	[82]
